# Online Peer Support for Youth at Higher Risk of or Living with HIV: A Qualitative Content Analysis

**DOI:** 10.1007/s10461-025-04677-z

**Published:** 2025-03-16

**Authors:** Alice M. Ma, Katherine A. Lewis, Mahnoor Wani, Camellia Liu, Sabrina Ghalambor, Rhitishah Yuva Raju, Curtis Wong, Dallas Swendeman, ATN CARES Study Team, Sue Ellen Abdalian, Elizabeth Arnold, Robert Bolan, Yvonne Bryson, Antwon Chaplin, W. Scott Comulada, Ruth Cortado, Catherine Donahue, Maria Isabel Fernandez, Risa Flynn, Jasmine Fournier, William Gertsch, Kelsey Ishimoto, Sergio Jimenez, Tara Kerin, Jeffrey Klausner, Jody Kussin, Sung-Jae Lee, Marguerita Lightfoot, Norweeta Milburn, Jasmine Mosafer, Aaron Moses, Debra A. Murphy, Karin Nielsen, Manuel A. Ocasio, Diana Polanco, Wilson Ramos, Cathy J. Reback, Panteha Hayati Rezvan, Mary Jane Rotheram-Borus, Wenze Tang, Yara Tapia, Demi Thomas, Stacey Urauchi

**Affiliations:** 1 Center for Community Health, Semel Institute for Neuroscience and Human Behavior, University of California Los Angeles, 760 Westwood Plaza, Los Angeles, CA 90024 USA; 2 Department of Population and Public Health Sciences, Keck School of Medicine, University of Southern California, 1845 N Soto St, Los Angeles, CA 90032 USA; 3 Department of Epidemiology & Population Health, Stanford School of Medicine, Stanford University, 291 Campus Drive, Li Ka Shing Building, Stanford, CA USA

**Keywords:** Online peer support, HIV, Youth, Sexual health, Risk reduction, Mobile health intervention

## Abstract

With the rise of technology-based interventions for HIV risk reduction among adolescents, research is needed to assess how youth participate in these strategies. We used qualitative content analysis to examine youth’s posts in an online peer support intervention through the Adolescent Trials Network (ClinicalTrials.gov NCT03134833, registered May 1, 2017) for youth at higher risk of or living with HIV. We analyzed 308 posts across 85 conversations related to sexual health from 144 peer support participants. Conversation topics included risk reduction, partner navigation, sexual activity, awareness/advocacy, and consent/harassment. Results may inform the design, adaptation, and implementation of digital peer support interventions.

## Introduction

In the United States, people aged 13–34 accounted for 56% of new HIV diagnoses in 2021 [1]. Youth are less likely to be aware of their HIV status and are less likely to engage in sexual health and safety practices, such as pre-exposure prophylaxis (PrEP) and condom use, compared to adults [2].

Peer support has been suggested to promote care engagement by improving resilience, cultivating coping skills, and promoting social norms for healthy behaviors [3]. Mobile health and social media advances point towards online peer communities as platforms to engage youth and promote linkage and adherence to HIV prevention and treatment [3]. Past studies investigating peer-led online communities and groups as intervention modalities have shown that individuals who participated in online posts about HIV prevention and testing were more likely to request an HIV testing kit than non-participating individuals [3]. Participants in similar studies have reported positive experiences and endorsed potential benefits for treatment adherence, stigma reduction, and mental and behavioral health [2, 4, 5].

Despite this, the specifics of how youth interact on peer support platforms for HIV prevention and what conversations they engage in have not been adequately explored. This information is needed to inform future implementation and scale adjustments. To address this gap, we analyzed posts from youth at higher risk of or living with HIV (YAHRLH) engaged in an online peer support forum within the context of a large, multi-site randomized controlled trial (RCT) for youth at higher risk of HIV [6] and two linked studies for youth living with HIV [7, 8]. The RCT study results indicated that youth at higher risk of HIV randomized to receive the peer support intervention coupled with coaching and automated text messaging had increased PrEP use sustained over a 24-month follow-up period [6]. Considering these promising results and looking towards intervention implementation, we aim to assess the content of conversations on the peer support forum to understand how youth engaged in this intervention modality.

## Methods

### Data Collection and Study Methods

Youth ages 14 to 24 years in Los Angeles, CA and New Orleans, LA were enrolled in one of three HIV prevention and treatment studies through the Adolescent HIV Medicine Trials Network (ATN) between 2017 and 2019. Participants included youth with acute (newly diagnosed) HIV infection (ATN 147; [7]), youth with established HIV infection (ATN 148; [8]), and youth at higher risk of HIV (ATN 149; [9]). Participants were screened and enrolled through community-based organizations and active social media outreach. Eligibility was based on HIV-positive status; sexual or gender minority status; or multiple risks of lifetime experiences of homelessness, incarceration or probation, mental health hospitalizations, substance abuse treatment, recent sexual risk behaviors, or illicit drug use [10].

These studies examined the efficacy of three interventions aimed at promoting youth’s engagement with the HIV prevention and treatment continua: automated text messaging and monitoring (AMMI), online and anonymous peer support groups (PS), and strengths-based coaching [10]. Youth at risk were randomly assigned to one of four intervention groups: (1) AMMI alone, (2) AMMI plus PS, (3) AMMI plus Coaching, or (4) AMMI plus PS plus Coaching (combined intervention). Participants from the AMMI plus PS intervention and the combined intervention are included in these results. Youth living with established HIV infection were randomized to AMMI only or stepped-care with PS as the second intervention offered after AMMI triggered after being virally unsuppressed (VL > 200) at a study follow-up. Youth newly diagnosed were offered all interventions. Participants were incentivized with $10 weekly to post three times per week for up to 16 weeks over 24 months of study follow-up [9].

Online peer support was hosted on muut.com, a platform similar to Reddit or Discord, where participants could post and respond to discussion board-style conversations under anonymous usernames. This platform was selected to address Institutional Review Board (IRB) concerns over other platforms that permit direct messaging between individuals. The platform organized content into conversations and posts, where each conversation is a collection of individual posts pertaining to that thread. The peer support boards were monitored by near-peer coaches who promoted engagement and corrected misinformation, as needed. Coaches were initially identified as coaches through their usernames but later changed their names to non-identifying usernames in response to youth advisory board feedback. The conversations were youth-centered and addressed topics relevant to participants in addition to HIV prevention.

### Analysis Methods

We used qualitative content analysis to analyze all conversations and posts over the course of the study [11]. Content analysis was used to effectively evaluate our large, diverse dataset in its entirety and systematically condense it to quantify and summarize aspects related to our main theme of sexual health. Qualitative content analysis allowed us to focus on a narrow topic while maintaining fidelity of coding and offering flexibility in allowing our coding frame to fit our data. All coding was conducted manually in Microsoft Excel and Google Sheets. Members of the coding team represented diverse identities with regard to gender, race/ethnicity, and sexual orientation. All coders completed training on qualitative methods and content analysis, which included information about reflexivity. An initial round of data reduction was conducted where each post and conversation was coded for relevance to sexual health, defined as any content related to the promotion of sexual health and/or the reduction of sexual risk. Because the data were organized as posts within conversations, all content analysis was conducted at both the conversation and post level. During data reduction, a conversation was coded as relevant to sexual health if any of the posts pertained to sexual health. When coding at the post level, each individual post was coded as either relevant or not relevant. Every post and conversation was individually coded blindly by three coders, and data were considered irrelevant if all coders agreed on irrelevance (i.e., if even one coder coded the post as relevant, it was retained in the reduced dataset). Following data reduction, a pilot coding round was conducted using 10% of the relevant dataset. The pilot coding frame was developed using a deductive approach, based on relevant sexual health risk reduction topics (PrEP, condom use, partner communication, etc.). Each post and conversation was coded independently by two coders and discrepancies were decided by a third coder. Conversations were coded based on their overall theme, while individual posts within those conversations could be assigned codes that either aligned with or differed from the conversation’s main theme. During pilot coding, the coding frame was assessed for consistency in coding between coders and modified to clarify points of confusion before a final coding frame was developed and used for the entirety of the sexual health dataset. The coding team met weekly to resolve any questions about code application. After all coding had been completed, STATA version 18 was used to generate summary statistics and frequencies for each code. Following the completion of this content analysis procedure, we returned to the conversations that had been excluded as irrelevant and reviewed them to develop an inductive list of non-sexual health-related conversation topics.

## Results

**Fig. 1:**
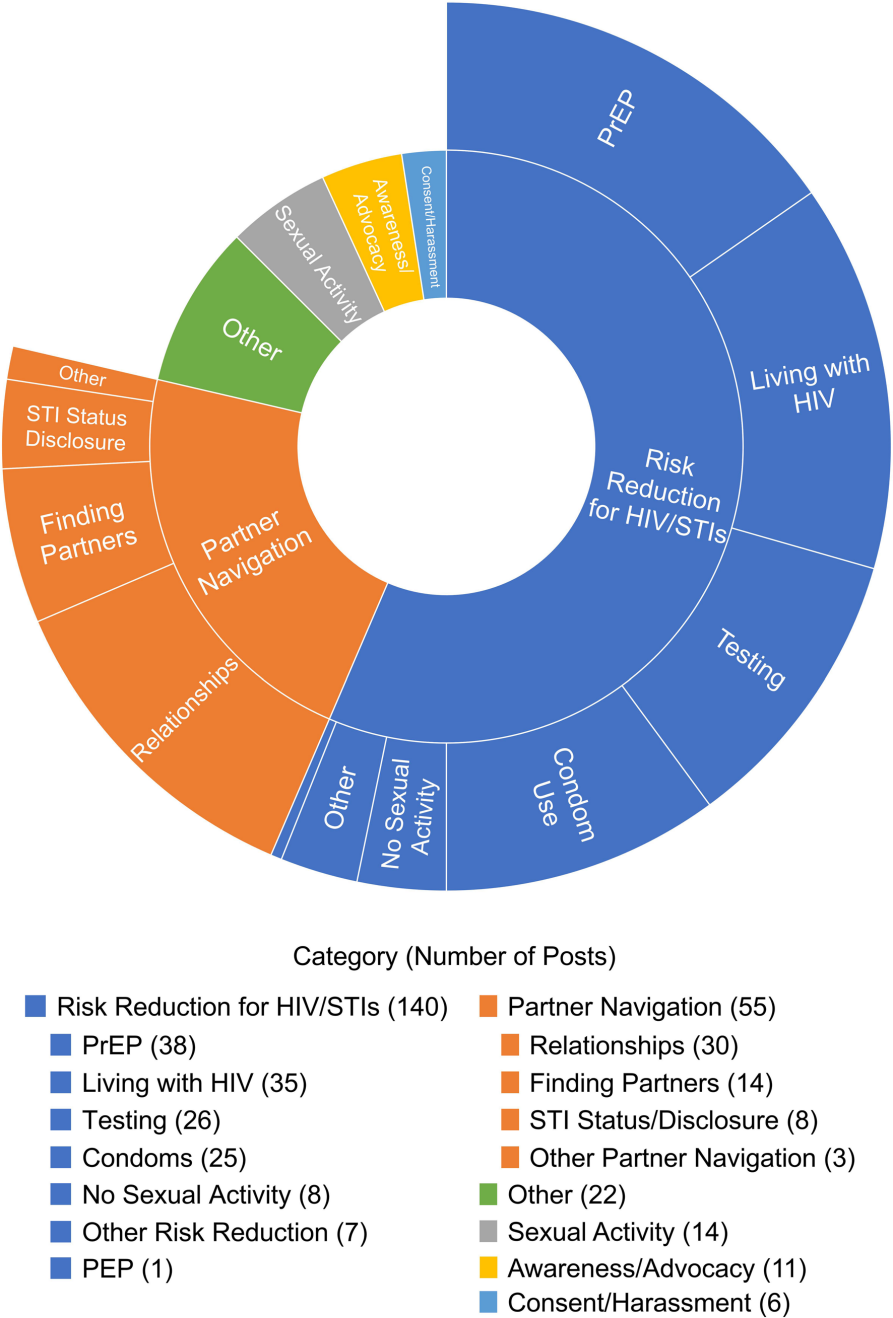
Pie chart of percent of posts per sexual health themes

Overall, 670 participants were assigned to or offered the peer support intervention. 15% completed only the baseline assessments and were not contactable for subsequent intervention activities, leaving 570 participants. Out of these, 144 (25.3%) engaged in 1433 conversations, making 5142 posts. A total of 85 conversations were related to sexual health consisting of 308 posts. When coding at the post-level, 248 individual posts were relevant to sexual health while 60 posts were coded as ‘not relevant’ but were made within conversations that were included in the sexual health relevant subset. This would include, for example, participants expressing agreement with each other or continuing a conversation with something not related to sexual health or sexual risk reduction. Figure 1 displays the frequency of each sexual health code in the analysis.

Each sexual health code is discussed separately below. Percentages are reported as the percentage of sexual health posts (n = 248) and the percentage of all sexual health relevant conversations (n = 85) for each included code.

1. Risk Reduction for HIV and STIs (140 posts (56.5%), 51 conversations (60.0%)): The most common sexual health code was Risk Reduction for HIV and STIs, with 140 posts spanning 51 conversations (56.5%). Of the total 85 conversations, 51 (60.0%) were coded at the conversation-level as being primarily related to risk reduction for HIV/STIs. These conversations focused on strategies to reduce the risk of contracting HIV and/or other STIs. This code included 6 subcategories: PrEP, Living with HIV, Testing, Condom Use, No Sexual Activity, Other Risk Reduction Strategies, and PEP.

1a. PrEP (38 posts, 10 conversations): Participants sought information on PrEP and discussed barriers to PrEP access. Discussions also included opinions on PrEP usage, such as discussions about side effects, comfort with and acceptability of the drug, and willingness to initiate PrEP use. For instance, some participants questioned whether they should start taking PrEP due to side effects.

Participant A: I don’t know whether i should take my prep pills or not because my friends tell me that its not worth taking them because of the side effects and the fact that there using us as ginnie pigs so i don’t know what to do should i take them i should i stop.

Participant B: Have you talked with your doctor about your concerns? I am planning on starting prep, but am waiting until I can get a reliable supply. But I am nervous about the side effects, and may stop taking it if they’re too troublesome.

Participant C: I have multiple friends using PREP who have never experienced negative side-effects. This pill is not using people as a test subject, it’s here to help prevent the spread of HIV. If you’re having sex, I definitely recommend it!

Participant A: No I haven’t talked to a doctor

Participant D: Talk to your doctor or someone at the LGBT Center! Prep is changing and saving lives in my opinion. I’ve never experienced any side affects, and I’ve been taking it for 3 years.

1b. Living with HIV (35 posts, 16 conversations): Participants shared personal experiences with disclosing HIV status, offered dating advice for those living with HIV, and discussed transmission risks. They also discussed insurance coverage of and access to HIV medications. For example, one participant sought advice on when they should communicate with their partners about their status.

Participant A: As a person who is HIV undetectable, when is a good time to let someone you know you are dating about your status?

Participant B: As soon as possible in my opinion

Participant C: I tend to tell people before I even meet them I’m not going to re-put myself in the closet just for dating or out of fear of someone’s lack of knowledge

1c. Testing (26 posts, 11 conversations): Participants exchanged information about the availability and accessibility of free HIV/STI testing centers in Los Angeles and New Orleans, shared resource links, and described personal testing experiences. Some participants talked about COVID-19’s impact on HIV testing availability.

Participant A: With COVID-19 occurring, does any one know any companies to get tested for free and won’t need like insurance. It’s been like 6 months since I have received a test. Thank you! :D

Participant B: AHF (Aids Health Foundation) does testing for free, no insurance needed. I would also suggest the LA LGBT center, they are also free.

Coach A: Planned Parenthood also provides STI/HIV testing regardless of insurance status.

1d. Condom Use (25 posts, 8 conversations): Participants shared experiences of asking partners to use condoms, dealing with partners who refuse to use condoms, and emphasizing the importance of protecting oneself during sexual encounters. For example, some participants asked for advice on how to bring up condom use to their partner.

Coach A: Have you ever had a conversation with someone you’re dating about using condoms? If yes, how did it go? If no, how would you bring up the topic?

Participant A: Yes. We were talking about when ever the time came when we decided to be together we would have to have use a condom. He gave the typical I don’t like them it doesn’t feel the same and don’t you love me? I just told him I do but neither does having a baby and I’m not ready to have a baby yet. Never feel pressured into doing something you don’t feel comfortable doing.

Coach B: Absolutely. Bring it up, be assertive. Using a condom benefits and protects both of y’all. Sounds like a win-win.

1e. No sexual activity (8 posts, 2 conversations): Some participants talked about strategies for staying safe, including abstaining from sexual activity and dating app use. This included conversations about decisions to not engage in sexual activity as a means of risk reduction, with some expressing contentment from their decision.

1f. Other Risk Reduction Strategies (7 posts, 4 conversations): These conversations focused on risk reduction but did not fit into other categories. Some participants expressed challenges and concerns related to sexual health during the COVID-19 quarantine period and stigma around STIs/HIV.

1 g. PEP (1 post, 0 conversations): One participant posted about seeking more information on people’s experiences using PEP. However, this conversation was not primarily about PEP use.

2. Partner Navigation (55 posts (22.2%), 14 conversations (16.5%)): The second most prevalent theme among participants was Partner Navigation. These conversations broadly focused on sexual and/or romantic partners. They can be further categorized into 4 subthemes: Relationships, Finding Partners, STI Status Disclosure, and Other Partner Navigation.

2a. Relationships (30 posts, 6 conversations): Many participants explored the complexities of forgiving a cheating partner and shared experiences with open relationships. For example, one participant expressed fear of infidelity and non-monogamy.

Participant A: I fear not being enough for my partner. I fear being left or cheated on because I’m not able to have sex every time he wants or because I’m not as good as someone else. I’m scared of not being sexy enough for not having a big enough ass. That I don’t have a perfect body. I’m scared that if I don’t give him the sexual attention he wants he’ll leave or ask for an open relationship

Participant B: Have you been able to talk with him about these fears? confidence might be the most sexy thing honestly. knowing what u got and what ur proud of will amplify it. just be sure and vocal about what YOU want.

Participant C: think about hook ups you’ve had where your partner was very confident. it makes a difference right?

2b. Finding Partners (14 posts, 4 conversations): Participants expressed discontent with dating apps and meeting people in person. For example, some participants revealed struggles to establish meaningful connections with people besides sexual encounters. Besides dating apps, a few participants discussed similar discontentment with finding partners in-person.

Participant A: Does anyone feel like it is difficult to make real connections with guys in LA? I can meet guys who want to hook up but whether I meet them at bars or in dating apps, every guy is striving for unrealistic standards in a partner. It makes it really difficult to have a satisfying conversation.

Participant B: Dude! For real, but for me personally. I know that I cannot dedicate myself to forming new relationships or friendships because my commitment to nursing school is hard to manage and give time to someone like that. I think a lot of people in LA tend to put work first and hustle now and worry about love later. Getting deep in conversation is really not an LA culture thing. It’s quite interesting to talk about. I also think the younger the guys, like myself, are stuck to the perfect movie romances. We want to experience that. I hope that make sense. I hope you do find someone soon!

Participant A: Thanks!

2c. STI status/disclosure (8 posts, 3 conversations): Many participants voiced concern about partner dishonesty regarding their STI status. These discussions predominantly centered on confronting or meeting the person they suspected of transmitting the STI. For example, some participants shared experiences of being lied to about a partner’s STI status.

Participant A: Has anyone ever been lied to by their sex partner about their status ?

Participant B: Yeah. My ex was cheating on me and I figured it out when I got tested again a few months into our relationship and I was positive for oral herpes all of a sudden after not having it the first time I got tested. He broke up with me without telling me about cheating and I asked if he had been exclusive to me since you can get herpes in so many nonsexually transmitted ways, but he wasn’t honest about it at the time (he told me later though).

Participant A: Yikes I’m so sorry about that that’s a pretty messed up thing to do

Participant B: it’s okay. It’s been almost four years since then now. I’m mostly over it. At first I was more upset about losing his friendship than anything but I’ve made WAYYY better friends since then so now I only miss the sex every once in a blue moon lol.

2d. Other Partner Navigation (3 posts, 1 conversation): In one conversation, participants recommended non-sexual activities to do with partners such as cooking.

3. Sexual Activity (14 posts (5.7%), 6 conversations (7.1%)): Participants discussed sexual experiences and preferences that were not framed within the context of sexual health or risk reduction. Participants most often discussed their own sexual experiences, with two conversations centered on experiences with topping and one conversation about threesomes. Two posts also included questions about sex, such as wondering about the normalcy of certain sexual desires. Participants also expressed feelings of sexual dissatisfaction or frustration from lack of sexual activity.

4. Awareness/Advocacy (11 posts (4.4%), 3 conversations (3.5%)): Participants shared information about advancements in HIV/AIDS research and treatment and raised awareness about World AIDS Day. Participants also expressed gratitude for the LGBTQ + community and how community advocacy helped them learn, destigmatize, and re-evaluate their relationship with and understanding of HIV. One conversation also focused on studies of how HIV infections have disproportionately impacted certain ethnicities, resulting in stigmatization within some communities and harmful stereotypes.

Participant A: I just want to say that I am thankful for the LGBTQ+ community for all their efforts to educate and help eradicate HIV infection. This year I have learned so much about HIV and it has really helped me curb the stigma I have had. I am advocating for a world where medication can reach all parts of the world and we can finally end the spread of HIV. #SpreadFactsNotFear

Participant A: I learned about the medication and the advances made. I got to fully comprehend what undetectable means. And honestly a big part of it was finding this YouTube channel called [de-identified creator name]. This is a vlogging based channel of [de-identified creator name] sharing his experience having HIV. He is very insightful about his journey and watching him really did away with a lot of stigma I had. There is a particular video where he goes to the doctor for his check up and there is a lot of insightful information given that really humanizes everything.

Coach A: This is great! Thank you for sharing. What sorts of things have you learned this year to change how you feel?

Participant B: We’ve definitely come really far in the past several years. And the fact that now medications are so good that they can prevent HIV and also make it so the virus is completely undetectable and not spreadable is incredible and I’m so grateful.

5. Consent/Harassment (6 posts (0.2%), 1 conversation (1.2%)): In one conversation, participants shared experiences of unwanted sexual contact in bars.

6. Other (22 posts (0.9%), 10 conversations (11.8%)): This includes any sexual health topics that do not fit into the codes listed above. Participants discussed societal views on sex and navigating discussions about sex in both public and private settings. Participants asked for tips and advice for topics ranging from general and sexual means of protection, small comforts in life, bringing up sex with parents, and how to stay sober. Other conversations falling under the umbrella of “other” included experiences within the study itself, the societal taboo of sex, thoughts on soulmates and relationships, and the gay going out scene.

### Non-Sexual Health Content

Besides the sexual health content described above, the remaining 4834 posts across 1347 conversations (94% of all posts) consisted of other conversations. Upon inductive review, non-sexual health conversations covered a very broad spectrum of topics. Health-related content included: mental health experiences, resources, and strategies; substance use, addiction, and recovery; physical health (sleep, diet, sickness, exercise); self-care and stress relief; and COVID-19 pandemic and lockdowns. Content related to participants’ lives and experiences included: work/employment, hobbies, housing, traveling, friendships/social relationships, pets, personal experiences with transitioning and/or coming out, experiences in the ATN-CARES study, and plans for the weekend or for extracurricular activities. Conversations about current events included the Black Lives Matter movement and activism, the 2020 presidential election, California wildfires, and the COVID-19 vaccine rollout. General conversation topics also included pop culture (movies, TV, books, music, sports), food and restaurants, holidays, weather, motivation and encouragement, and astrology.

Participant A: I don’t have medical insurance, but I’m very interested in seeking affordable therapy sessions. Does anyone know of a potential referral or where I’d begin to look?

Participant B: You can try an LGBT center, my sister also gave me this resource a while ago: [de-identified website link] . They have affordable therapy for lgbt folks

Participant A: I’m definitely going to check that out, thanks for the rec!

Participant C: Look up some online!

Participant D: St Johns WellChild and Family center on [de-identified street name] is amazing. It’s free if you can’t afford it or have no insurance.

## Discussion

In this analysis, we examined the content of peer support discussion posts by YAHRLH to better understand how youth engaged in this intervention. The majority of posts in the peer support forum (94%) focused on non-sexual health topics, such as current events, pop culture, and employment. Sexual health-related posts represented 6% (n = 308) of the posts across 85 conversations. The peer support intervention did not restrict discussions to HIV or sexual health. Coaches initiated broader topics of conversation to drive engagement and build community, and participating youth also initiated a wide variety of other topics. Despite this, participants engaged in meaningful sexual health and risk reduction conversations ranging from personal inquiries about practices, such as PrEP use and disclosing HIV/STI status to partners, to expressions of interest in advocacy work, and referrals and recommendations for service providers.

Our finding of a limited percentage of sexual health discussions is in line with other social network-based HIV studies in which participants were not obligated to engage in conversations about HIV-related subjects [3, 12]. For example, in a social networking study focused on HIV education, only 29% of the total discussions among participants were pertinent to HIV-related themes [3]. Despite the small percentage of related discussions, previous study results consistently highlight the acceptability of social media-based interventions for HIV risk reduction [3].

Several studies have highlighted the positive impact of participating in HIV-related discussions within peer support contexts, leading to the adoption of risk reduction behaviors, including increased HIV self-testing and enhanced treatment adherence [2, 3, 12]. Participants in our study who received a combined intervention (coaching + peer support + texting) demonstrated a sustained increase in PrEP uptake over the two-year follow-up period compared to those in other intervention groups [6]. This analysis contributes to this literature by providing more detail on how youth engaged with the peer support intervention. This provides insight into how the peer support intervention may have contributed to the observed increase in PrEP use over the follow-up period, where peers could address pharmaceutical mistrust and side effect concerns, share their personal experiences and community benefits, and suggest PrEP care providers.

While our study employed rigorous qualitative content analysis methods, certain limitations warrant consideration. The nature of content analysis introduces an inherent challenge of subjective interpretation regarding what qualifies as a sexual health-related post or a post that fits into any of the codes within the coding frame. To address this challenge, we used two blinded coders and a tie-breaker coder when needed. Another limitation is that there was not universal participation by all youth assigned to receive the intervention. Online peer support is not universally acceptable or perceived to be a valuable use of time, but may be valuable to some youth, indicating the value of multiple intervention strategies including automated text messaging and coaching. The widely accessible nature of online peer support was further exemplified during the COVID-19 pandemic as participants continued to maintain these online conversations despite quarantine and isolation periods. However, Muut is not commonly used by youth and there were challenges in getting participants to create accounts. In real-world practice, a platform like Discord that many youth already use may be more acceptable and accessible, resulting in higher engagement.

Future research should expand on these results by exploring whether participation in this or a similar online peer support intervention is associated with increased sexual health promotion behaviors, improved partner communication/navigation, or increased engagement with mental health systems as discussed in the peer support boards. For example, in our analysis, participants shared resources for accessible, non-stigmatizing testing and therapy services. Future research could explore whether there is an association or a dose–response relationship between peer support board participation and engagement with relevant services. Future work should also focus on optimizing and tailoring these programs to address unique needs and challenges of diverse adolescent populations. Our results also have implications for public health practice. Online peer support boards could help public health practitioners reach youth who do not have access to more traditional in-person services, such as those living in rural areas and those with transportation barriers. This intervention modality could also provide opportunities for peer support and community-building among LGBTQ + youth or others who are part of minoritized groups who are living in environments without large, accepting communities of peers.

## Conclusion

Online peer support programs are a promising area for HIV risk reduction among youth. Our study offers insight into the types of discussions that may hold value for youth in the context of peer support for HIV prevention and treatment, including a wide range of topics and meaningful conversations related to sexual health and risk reduction. These results may be used to inform ongoing implementation and scale-up efforts to improve adolescent health outcomes.

## Data Availability

Data will be made available upon reasonable request. To request data, please email the corresponding author.
